# EDXRF dataset of late classic Maya obsidian from the North Western Maya Lowlands^☆^

**DOI:** 10.1016/j.dib.2018.03.046

**Published:** 2018-03-17

**Authors:** Flavio G. Silva de la Mora

**Affiliations:** UC Berkeley, United States

## Abstract

The database includes the results from a geochemical and behavioral analysis of obsidian artifacts and debitage in the article entitled “Obsidian Procurement and Distribution in the Northwestern Maya Lowlands during the Maya Classic, a Regional Perspective” (Silva de la Mora, 2018) [3]. The information includes the statistical summaries of formal attributes used in the analysis: weight, diameter, thickness, length, width, formal attributes, type of artifact, context, and provenance for each artifact. The database illustrates the results from the EDXRF analysis of major, minor and trace element quantification, including the geologic control: RGM-1 (U.S.G.S. Rhyolite, Glass Mountain).

**Specifications Table**

**Table:** 

Subject area	*Archaeology, Anthropology*
More specific subject area	*Classic Maya Archaeology*
Type of data	*File, Figures, Tables*
How data was acquired	Thermo Scientific ARL QUANT’X Energy-Dispersive XRF Spectrometer Geoarchaeological Laboratory at UC Berkeley
Data format	*EDXRF raw data, analyzed, and summary of data of the behavioral attribute analysis*
Experimental factors	*Artifacts were cleaned with water by hand (no mechanical force was used)*
Experimental features	*Flintknapping was done by the author to understand the lithic scars*
Data source location	*Chiapas and Tabasco in Mexico and (Upper NW limit) Lat: 17.898426° Long: − 92.495786°, UTM Zone 15Q E553408.45 N1979020.05 (Lower SE limit) Lat 16.874668° Long − 90.78158°, UTM Zone 15Q E736341.45 N1867018.93*
Data accessibility	*The data generated is accessible within this article.*

**Value of the data**

• The data summarizes the statistical results from the behavioral analysis of all the artifacts analyzed.
• The data illustrates the raw results from the EDXRF study of each artifact, which can be compared and used for similar studies.
• The data complements similar comparative studies with analogous methodologies and techniques.

## 1 Data

**Fig. 1:**
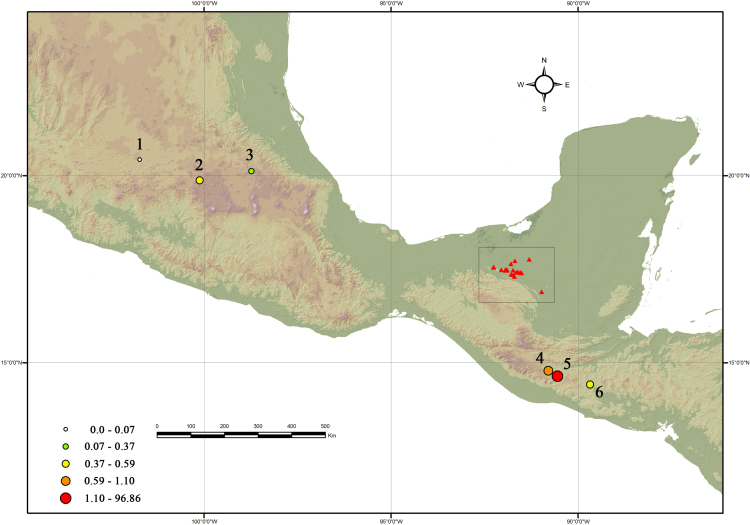
Location of study region with percentages of obsidian by source: 1) Penjamo, 2) Zaragoza, 3) Pachuca, 4) Jilotepeque, 5) El Chayal, 6) Ixtepeque.

The Supplementary Tables 1–9 summarize and outline the results from the behavioral analysis [3]. The Fig. 1 shows the proportion of obsidian by source in study. The Appendix A has the raw data from the geochemical analysis of EDXRF of trace elements in ppm (parts per million) [3].

## 2 Experimental design, materials and methods

Every artifact was analyzed using a behavioral analysis of the formal attributes and physical characteristics [2]. The tables illustrate the results from the formal analysis that includes length, width, thickness and weight for each artifact, separated by type of tool (Supplementary Table 1–9). The supplementary dataset exhibits the results of the element compositional analysis using EDXRF and the controls of a known USS Geochemical Reference standard – Rhyolite, Glass Mountain (RGM-1) [1]. The acronyms for the elements and their meaning are the following:

**Ti**: Titanium

**Mn**: Manganese

**Fe**: Iron

**Zn**: Zinc

**Rb**: Rubidium

**Sr**: Strontium

**Y**: Yttrium

**Zr**: Zirconium

**Nb**: Niobium

**Pb**: Lead

**Th**: Thorium

**B1S1**: nomenclature given to each artifact: B (Bag number) and S (Sample number).

**RGM1**: Rhyolite, Glass Mountain Sample 1.
