# Supplementary material for: EDXRF dataset of late classic Maya obsidian from the North Western Maya Lowlands
Source: Data Brief. 2018 Mar 17;18:492–5. doi: 10.1016/j.dib.2018.03.046 (PMC5996267; doi:10.1016/j.dib.2018.03.046)
Supplement: Supplementary file 1 — Supplementary material [file mmc1.pdf]

Conflicts of Interest Statement

Manuscript title:

Attribute analysis results and EDXRF dataset of Late Classic Maya

obsidian from the North Western Maya Lowlands

The authors whose names are listed immediately below certify that they have NO affiliations with or involvement in any organization or entity with any financial interest (such as honoraria; educational grants; participation in speakers' bureaus; membership, employment, consultancies, stock ownership, or other equity interest; and expert testimony or patent-licensing arrangements), or non-financial interest (such as personal or professional relationships, affiliations, knowledge or beliefs) in the subject matter or materials discussed in this manuscript.

Author names:

Flavio G. Silva de la Mora

The authors whose names are listed immediately below report the following details of affiliation or involvement in an organization or entity with a financial or non-financial interest in the subject matter or materials discussed in this manuscript. Please specify the nature of the conflict on a separate sheet of paper if the space below is inadequate.

Author names:

This statement is signed by all the authors to indicate agreement that the above information is true and correct (*a photocopy of this form may be used if there are more than 10 authors*):

Author's name (typed)	Author's signature	Date
Flavio G. Silva	Flavio G. Silva	Feb 2, 2018
